# Definition and validation of the nursing diagnosis label “wish to die”: a research protocol

**DOI:** 10.1186/s12912-024-01707-4

**Published:** 2024-01-12

**Authors:** Cristina Monforte-Royo, Blanca Goni-Fuste, Iris Crespo, Denise Pergolizzi, Leandra Martin-Delgado, Pilar Fuster, Mercedes Bellido-Perez, Joaquín Tomás-Sábado, Andrea Rodríguez-Prat

**Affiliations:** 1 Nursing Department, School of Medicine and Health Sciences, Universitat Internacional de Catalunya, Josep Trueta s/n, Sant Cugat del Vallès, Barcelona, 08195 Spain; 2 Basic Science Department, School of Medicine and Health Sciences, Universitat Internacional de Catalunya, Josep Trueta s/n, Sant Cugat del Vallès, Barcelona, 08195 Spain; 3 School of Medicine and Health Sciences, Universitat Internacional de Catalunya, Josep Trueta s/n, Sant Cugat del Vallès, Barcelona, 08195 Spain; 4 Primary Health, Institut Català de la Salut, Barcelona, Spain; 5 Faculty of Humanities, Universitat Internacional de Catalunya, Josep Trueta s/n, Sant Cugat del Vallès, Barcelona, 08195 Spain

**Keywords:** Nurses, Nursing, Nursing diagnosis, Wish to die, Wish to hasten death, Euthanasia, Palliative care, Primary health care, Nursing taxonomy, NANDA, Defining characteristics, Related factors

## Abstract

**Background:**

Euthanasia has been incorporated into the health services of seven countries. The legalisation of these practices has important repercussions for the competences of nurses, and it raises questions about their role. When a patient with advanced disease expresses a wish to die, what is expected of nurses? What are the needs of these patients, and what kind of care plan do they require? What level of autonomy might nurses have when caring for these patients? The degree of autonomy that nurses might or should have when it comes to addressing such a wish and caring for these patients has yet to be defined. Recognising the wish to die as a nursing diagnosis would be an important step towards ensuring that these patients receive adequate nursing care.

This study-protocol aims to define and validate the nursing diagnosis *wish to die* in patients with advanced disease, establishing its defining characteristics and related factors; to define nursing-specific interventions for this new diagnosis.

**Methods:**

A prospective three-phase study will be carried out. Phase-A) Foundational knowledge: an umbrella review of systematic reviews will be conducted; Phase-B) Definition and validation of the diagnostic nomenclature, defining characteristics and related factors by means of an expert panel, a Delphi study and application of Fehring’s diagnostic content validation model; Phase-C) Definition of nursing-specific interventions for the new diagnosis. At least 200 academic and clinical nurses with expertise in the field of palliative care or primary health care will be recruited as participants across the three phases.

**Discussion:**

The definition of the wish to die as a nursing diagnosis would promote greater recognition and autonomy for nurses in the care of patients who express such a wish, providing an opportunity to alleviate underlying suffering through nursing-specific interventions and drawing attention to the needs of patients with advanced disease. The new diagnosis would be an addition to nursing science and would provide a framework for providing care to people with advanced disease who express such a wish. Nurses would gain professional autonomy about identifying, exploring and responding clinically to such a wish.

**Supplementary Information:**

The online version contains supplementary material available at 10.1186/s12912-024-01707-4.

## Background

Euthanasia or medical assistance in dying (MAiD) has now been incorporated into the health services of seven countries (Netherlands, Belgium, Luxembourg, Colombia, Canada, New Zeeland and Spain). The legalisation of these practices has important repercussions for the competences of health professionals. For nurses, it raises questions about what their role should be when a patient expresses a wish to die, which may or may not lead to a formal request for euthanasia or MAiD. What is expected of nurses when a patient with advanced disease expresses such a wish? What are the needs of these patients, and what kind of care plan do they require? What level of autonomy might nurses have when caring for these patients? Although these questions remain largely unanswered, we do know that these patients have specific needs and that they require nursing care. Over the past decade, several studies have analysed the wish to die or to hasten death and have sought to define its characteristics and related factors, and to a lesser extent to consider how such a wish may be explored and addressed [1–6]. To date, however, the degree of autonomy that professional nurses might or should have when it comes to addressing such a wish and caring for these patients has yet to be defined. In our view, therefore, recognising the wish to die as a nursing diagnosis would be an important step towards ensuring that these patients receive adequate nursing care.

Nursing diagnoses are a defining feature of nursing science [7]. The use of standardised nursing language and terminology provides a reference framework for describing and defining the phenomena and situations that nurses encounter in their autonomous clinical practice [8]. Among the benefits of having a standardised nursing language are: a) better care [9], b) increased visibility for nursing practice [10], c) facilitating the incorporation of information into electronic health records [11], d) promoting critical thinking, e) improved intra- and interprofessional communication, f) helping to ensure individualised care, g) the possibility of comparison, thus enabling the effectiveness of nursing care to be evaluated, and h) providing a stronger foundation for the nursing profession. In sum, a standardised nursing language offers a universal and solid conceptual basis for describing the host of phenomena and situations that nurses may encounter when caring for patients [12].

One of the most well-known diagnostic taxonomies in nursing is NANDA-I [13, 14], which is recognised and widely used in both clinical and academic settings [15]. A nursing diagnosis is defined in NANDA-I as “a clinical judgment concerning a human response to health conditions/life processes, or vulnerability for that response, by an individual, family, group, or community”, and it may be used to identify care outcomes and plan nursing-specific interventions [13]. NANDA-I is the only terminology that provides diagnostic indicators to support nurses’ clinical reasoning, and which considers care needs and nursing interventions. From this perspective, recognising and validating the wish to die as a nursing diagnosis would enable nurses to participate both in the identification of this phenomenon and in the planning and provision of care for patients who express such a wish, thereby providing a platform for autonomous nursing interventions.

### Conceptualisation of the wish to die

Patients with advanced disease may experience suffering as they try to come to terms with their illness and the end of life. This suffering can have a variety of causes (physical, psychological, etc.), and it leads some people to express a wish or desire to die (wish and desire are used as synonyms). This wish can emerge naturally and spontaneously in the context of the disease, without there being an underlying psychiatric disorder. The desire to die may manifest as a wish to hasten death (WTHD), planning for suicide or even attempts at suicide [16].

Within the field of psychiatry, a number of models have been proposed to guide suicide theory and prevention. According to the three-step theory [17], the WTHD would emerge – step 1 – when the level of psychological pain/suffering that is experienced is such that the person envisages no solution or possibility of improvement (i.e. they experience hopelessness). In step 2, the wish intensifies and progresses toward an active wish to die when the person feels that their connectedness to life (to family, friends) is absent or is less strong than their pain and sense of hopelessness. Finally, in step 3, the person has the capacity to attempt suicide (a capacity that depends on both dispositional and practical factors). From this perspective, professionals with responsibility for providing end-of-life care need to be attentive to the psychological and social resources that are available to a person with advanced disease so as to prevent, as far as possible, the emergence of a wish to die.

The fact that someone with advanced disease expresses a desire to die or WTHD does not necessarily mean that they wish to take action towards ending their life [4, 5]. People with suicide ideation obviously have a wish to die, but not all those who have thoughts of dying are actively considering suicide. According to Vehling et al. [16], the wish to die in people with advanced disease exists on a continuum, and only at one extreme will it be accompanied by suicide plans. Consequently, it is vital that the wish to die is detected in the early stages so that the person can be offered help to relieve their suffering, the ultimate aim being to prevent them from taking their life.

In 2016, a group of experts in the field of end-of-life care [1] published an international consensus definition of the WTHD in the context of advanced disease. The WTHD was defined as follows: *a reaction to suffering, in the context of a life-threatening condition, from which the patient can see no way out other than to accelerate his or her death. This wish may be expressed spontaneously or after being asked about it, but it must be distinguished from the acceptance of impending death or from a wish to die naturally, although preferably soon. The WTHD may arise in response to one or more factors, including physical symptoms (either present or foreseen), psychological distress (e.g. depression, hopelessness, fears, *etc*.), existential suffering (e.g. loss of meaning in life), or social aspects (e.g. feeling that one is a burden)*.

From the nursing perspective, it is essential to understand the different ways in which a wish to die may be expressed, including its most extreme manifestation in the form of an explicit request for MAiD, so as to be able to establish a care plan that is capable of responding to a person’s suffering before it becomes so unbearable that they are driven to action.

### Prevalence, assessment and related factors with the wish to die

Various instruments have been developed to assess the wish to die or WTHD. Estimates for the proportion of patients with advanced disease who experience such a wish range between 1.5% and 37.8%, depending on the instrument used and the characteristics of the sample surveyed [18].

With the aim of helping professionals not only to detect and quantify the wish to die in clinical practice but also to gain an idea of how often patients experience such a wish and how intense these wishes are, Porta-Salas et al. [19] developed a short semi-structured interview called the *Assessment of the Frequency and Extent Desire to Die* (AFEDD). When evaluating the AFEDD they found that patients with advanced disease considered that it was important and helpful to discuss this issue (even if they personally did not have a such a wish), and also that doing so was not upsetting in the majority of cases. This suggests that proactively exploring the wish to die with an instrument such as the AFEDD can help to understand more about a patient’s experience and, possibly, to identify sources of suffering that may be amenable to intervention.

As regards related factors, research has found a positive relationship between the wish to die and physical symptoms, functional impairment, the feeling of being a burden, lack of social support, depressive symptoms, hopelessness, poor quality of life, loss of perceived dignity, loss of control (self-efficacy) and loss of meaning in life [2, 6, 20, 21]. All these factors are reflected in the aforementioned consensus definition of the WTHD [1], highlighting the importance of its assessment and identification so as to implement adequate care plans.

In addition to related factors, some authors have explored the meaning of the wish to die from the perspective of patients who express it. In this context, the meta-ethnography by Monforte-Royo et al. [4] concluded that the WTHD is best seen as a reaction to multidimensional suffering and that, for people with advanced disease, it can have multiple meanings that do not necessarily imply a genuine desire to end one’s life. Based on their review and synthesis, these authors proposed an explanatory model of the WTHD, comprising six themes: 1) the presence of physical, psychological and spiritual suffering; 2) loss of self, resulting from the combined effect of the different losses that the person experiences (i.e. loss of function, of meaning in life, of perceived dignity, of control); 3) fear, both of death itself and of the dying process. The person therefore experiences hopelessness and intense emotional distress, and in response the WTHD may emerge: 4) as a wish to live but not in this way; 5) as a way of ending suffering; and 6) as a kind of control over one’s life, what the authors referred to as ‘having an ace up one’s sleeve just in case’.

### The nursing response to a wish to die

To the best of our knowledge, there are no studies that specifically consider how nurses might respond to a patient who expresses a wish to die, although there is research on the role of nurses in relation to euthanasia or MAiD [22–25]. In most cases nurses are excluded from the decision-making process, which generally involves only the patient and physician [26–28], but the experience of providing care in this context may nonetheless produce numerous moral and ethical dilemmas [29]. Even if professionals are aware of how much a patient is suffering, the request for euthanasia can leave them feeling that they are failing in their duty to meet the person’s care needs [24, 30].

Ethical dilemmas, insufficient training in how to relate to patients who express a wish to die [31] and the belief that dealing with this issue is not the responsibility of nurses are all factors that may lead to missed nursing care [32]. In this respect, the definition of a nursing diagnosis for the *wish to die* would raise awareness within the profession and help to focus attention on improving the care that is offered to patients who express such a wish.

## Methods / design

This protocol aims to define and validate the nursing diagnosis *wish to die* in patients with advanced disease, establishing its defining characteristics and related factors, and to define nursing-specific interventions for this new nursing diagnosis.

According to the American Society of Clinical Oncology, the European Association for Palliative Care and the Spanish Society of Palliative Care, advanced illness is defined as a progressive and incurable illness, with no apparent and reasonable possibility of response to specific treatment and where there are numerous intense, multiple, multifactorial and changing problems or symptoms that produce a great emotional impact on the patient, family and healthcare team, closely related to the presence, explicit or not, of death and with a prognosis of less than 6 months of life.

**Fig. 1:**
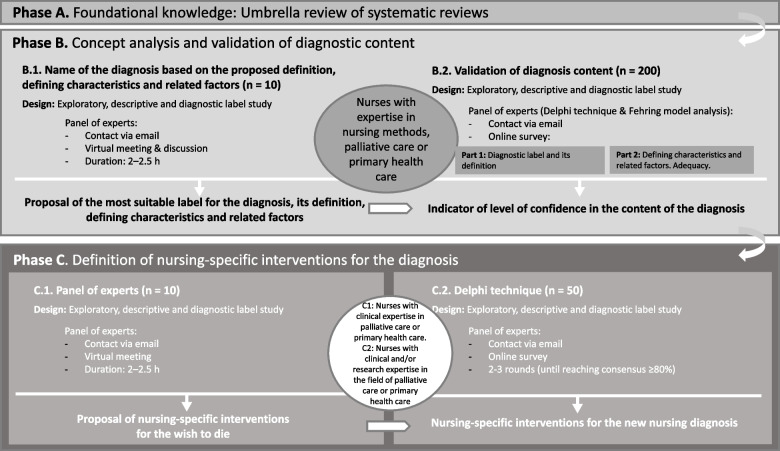
Figure legend: methodological research design

We will conduct a prospective study consisting of three phases: A) Establishing the conceptual foundation through an umbrella review of systematic reviews; B) Concept analysis involving a panel of experts, followed by use of the Delphi technique and application of Fehring’s [33] diagnostic content validation (DCV) model to reach a consensus; C) Definition of nursing-specific interventions for the new diagnosis, once again involving a panel of experts and use of the Delphi technique. Figure 1 shows a summary graphic of the three proposed phases.

### Phase A: Foundational knowledge

#### Design

We will conduct an *umbrella review* of all published systematic reviews of research and primary studies (not included in the systematic reviews) into the wish to die or WTHD among people with advanced disease, the aim being to analyse all the evidence regarding definitions of the WTHD, its aetiology and related factors, its assessment, and clinical interventions for responding to it. This review will serve to establish the conceptual framework that will underpin all subsequent phases of the research.

#### Data collection and data analysis

A search strategy will be designed and applied to the following databases: PubMed, CINAHL, PsycINFO and Web of Science. Articles for inclusion in the review will be selected in accordance with PRISMA guidelines [34].

After selecting articles for inclusion in the review, we will evaluate their quality, create a data extraction template and carry out an analysis and integrative synthesis of the research findings. The outcome of the review process will provide the conceptual framework for developing the nursing diagnosis and allow us to draw up a preliminary definition and set of defining characteristics and related factors, which will then be subject to concept analysis and content validation in Phase B of the research.

### Phase B: Concept analysis and validation of diagnosis content

#### B.1. Concept analysis: name of the diagnosis (the diagnostic label) based on the preliminary definition

##### Design

Exploratory, descriptive, and diagnostic label study involving a panel of experts. A panel of experts will analyse the proposed definition of the diagnosis and consider its type (syndrome or risk diagnosis) (see rationale in Additional file).

##### Sample/participants

A convenience sample of 8–10 participants [35] will be recruited from several countries from America, Australia and Europe, countries with a good palliative care development (with or without euthanasia or MAiD legalized). They will all be nurses with expertise in (a) nursing methods and/or (b) palliative care in a variety of settings (e.g. acute care hospital, outpatient clinic, domiciliary care and home support, long-stay care facility), and (c) primary health care nurses. This will ensure heterogeneity in the sample and will enrich the discourse. The rationale for focusing on palliative care is that patients here are more likely to express a WTHD than is the case in other settings [36].

##### Inclusion criteria

(a) For experts in nursing methods: at least 5 years experience of teaching nursing methods. (b) For experts in palliative care and primary health care nurses: at least 10 years experience in this field. For all participants: English speaker language, ability to communicate and express a reasoned opinion, extensive knowledge of and interest in the topic, scientific attitude (basing decisions on facts and data), intrinsic motivation (without need for external forms of reward) and signing informed consent. Professionals who declare any type of conflict of interest will be excluded.

##### Data collection and data analysis

Potential participants will be sent an email inviting them to form part of an expert panel and explaining its purpose, the tasks involved and the estimated time commitment (2–2.5 h). The plan is to use a virtual meeting platform (such as Zoom or Google Meet), the Google Jamboard and Google Forms to work on the proposed content and to classify and prioritise information.

The work of the expert panel will involve the following stages: (1) generation of ideas, (2) discussion, (3) summing up and conclusions, and (4) classification and prioritisation. The steering committee (research team expert in wish to die) will propose the first definition of the nursing diagnosis. This initial definition will be crucial for generating new ideas (stage 1) and to discuss. Discussion-analysis will be the basis of the nominal group. The objective of the nominal group will be to classify and prioritize information generated.

The labels proposed by the experts for the diagnosis will be categorised, and this, together with the analysis of ‘contextual units’, will enable a consensus to be reached regarding the most suitable label for the diagnosis, its definition, defining characteristics and related factors. This information will then be collated in the form of a questionnaire comprising two sections: 1) The diagnostic label and its definition, and 2) Defining characteristics and related factors. This questionnaire will be used for the validation of diagnosis content in phase B.2.

#### B.2. Validation of diagnosis content

##### Design

Exploratory, descriptive, validation study using Fehring’s [33] DCV model to determine the extent to which the defining characteristics and related factors are representative of the diagnosis.

##### Sample/participants

A convenience sample will be recruited comprising both academic and clinical nurses with expertise in areas of relevance to the diagnosis (i.e. nursing methods experts, palliative care or primary health care nurses) from America, Australia and Europe (continents with countries with and without euthanasia legalized). Participants in phase B.1 may also take part in this phase (B.2). Although Fehring [33] suggests that around 50 experts are sufficient for application of the DCV model, Nunnally and Bernstein [37] argue that greater reliability is obtained with a sample of 200, and accordingly this is the number we will recruit for this phase of the study.

##### Inclusion criteria

Same as in B.1.

##### Data collection

Potential participants will be sent an email inviting them to take part in a Delphi study and explaining its purpose, the tasks involved and the approximate time commitment. Participants will be recruited through snowball sampling of academic and clinical nurses in palliative care and primary health care. Experts who agree to participate will be asked to respond to an online questionnaire (hosted using Survey Monkey or Google Forms). The first part of the questionnaire will concern the diagnostic label and its definition; this will be broken down into several items, and in each case the task for experts will be to rate their agreement with the item statement. Two or three Delphi rounds will be conducted, with consensus ≥ 80% being required for an item to be accepted.

The second part of the questionnaire will list the defining characteristics and related factors for the diagnosis. This part of the questionnaire will be answered just once. The task for each expert will be to rate the extent to which each defining characteristic and related factor is, in their view, representative of the diagnosis, using the 5-point scale described in Fehring’s DCV model: 1 = not at all representative of the diagnosis; 2 = very little representative; 3 = somewhat representative; 4 = considerably representative; and 5 = very representative. Also as in Fehring’s model, weighted ratios will then be calculated for the ratings given to each defining characteristic and related factor, as follows: 0 = 1; 0.25 = 2; 0.50 = 3; 0.75 = 4; and 1 = 5.

##### Data analysis

The data will be analysed by two members of the research team.

For part 1 of the questionnaire (the diagnostic label and its definition): Questionnaire responses (ratings) will be entered into Microsoft Excel spreadsheets and the degree of consensus (weighted targeted agreement) for each statement will be calculated using the algorithm proposed by Tastle and Wierman [38]. Up to three Delphi rounds will be conducted, until consensus ≥ 80% is reached.

For part 2 of the questionnaire (defining characteristics and related factors): The mean weighted ratio will be calculated for each of the defining characteristics and related factors. Those with a ratio of .80 or higher will be considered as ‘major’ and as highly representative of the diagnosis, while those with a ratio less than .80 but greater than .50 will be classed as ‘minor’. Defining characteristics and related factors with a ratio less than .50 will be considered unrepresentative and will not be included.

Finally, a total DCV score will be calculated by summing the mean ratio scores and dividing by the total number of defining characteristics and related factors that have been retained. This score, which ranges between 0 and 1, will provide an indication of overall confidence in the content of the new diagnosis.

### Phase C: To define nursing-specific interventions for the new diagnosis

#### Design

Exploratory, descriptive and consensus study involving a panel of experts and the Delphi technique.

#### C.1. Panel of experts

To identify nursing-specific interventions for the new nursing diagnosis.

##### Sample/participants

Purposive sampling will be used to recruit 10–12 nurse experts in palliative care or primary health care who meet the following inclusion criteria: more than 10 years experience in palliative care or primary health care, English speaker language, ability to communicate and express a reasoned opinion, and signing informed consent. Participants will be recruited from different countries with or without euthanasia or MAiD legalized from America, Australia and Europe (continents with countries with and without euthanasia legalized) and a variety of palliative care settings (e.g. acute care hospital, outpatient clinic, domiciliary care and home support, long-stay care facility) so as to ensure heterogeneity in the sample. Experts who participated in phases B.1 and B.2 will also be eligible for this phase.

##### Data collection and data analysis

Potential participants will be sent an email inviting them to form part of an expert panel and explaining its purpose, the tasks involved and the estimated time commitment (2.5–3 h). The plan is to use a virtual meeting platform, data collection and data analysis as described for phase B.1. If consensus is not reached due the cultural, organizational or policy differences among participants, we will be open to carry out more than one expert panel. Theecisionns reached by the expert panel will be collated in the form of a questionnaire setting out the proposed nursing interventions for patients with advanced disease who express a wish to die. This questionnaire will then be used for the Delphi study in the next phase (C.2).

#### C.2. Delphi study

To reach an expert consensus regarding nursing-specific interventions for the new diagnosis.

##### Sample/participants

Purposive sampling will be used to recruit an international panel of 50 nurse experts in the wish to die among patients in palliative care or primary health care, who must fulfil at least one of the following inclusion criteria: (a) nurses, authors of published articles research in wish to die; (b) nurses with more than 5 years professional experience of palliative care or primary health care; (c) nurse researchers in the field of palliative care or primary health care from countries with or without euthanasia or MAiD legalized from America, Australia and Europe (continents with countries with and without euthanasia legalized). We will use English language. In order to ensure a high-quality Delphi process, we will recruit panellists from various countries with or without euthanasia or MaiD legalized and with experience in different palliative care settings (e.g. acute care hospital, outpatient clinic, domiciliary care and home support, long-stay care facility).

##### Data collection

Potential participants will be sent an email inviting them to take part in a Delphi study and explaining its purpose, the tasks involved and the approximate time commitment. Experts who agree to participate will be asked to respond to an online questionnaire (hosted using Survey Monkey or Google Forms). Two or three Delphi rounds will be conducted, with consensus ≥ 80% being required for a nursing intervention to be accepted.

##### Data analysis

Data will be analysed by two members of the research team. Questionnaire responses (ratings) will be entered into Microsoft Excel spreadsheets and the degree of consensus (weighted targeted agreement) for each statement will be calculated using the algorithm proposed by Tastle and Wierman [38].

### Ethical considerations

The study will be conducted in accordance with established codes of research ethics. The research protocol obtained the approval of the ethics committee of the Universitat Internacional de Catalunya. Participation will be entirely voluntary, and no remuneration will be offered. All data will be stored securely, and participants will be required to sign informed consent for their data to be used for research purposes. Three members of the research team will be responsible for analysing and coding the information obtained through the expert panels, and only they will have access to this data. Only the principal investigator will know the identity of experts who agree to participate in the panels. The responses of participants in the Delphi studies will be anonymised, and only two members of the research team will have access to the full data set.

### Rigour

The researchers behind this project are a close-knit team with combined experience in palliative care, nursing and systematic reviews. Specifically, the team comprises: 1) three internationally recognised researchers on the wish to die among patients with advanced disease; 2) 75% of its members are nurses with expertise in relation to standardised nursing language and terminology; 3) two researchers are nurses with experience of validating nursing diagnoses using Fehring’s [33] model; and 4) three of the team members teach nursing methods on university courses. In addition, the principal investigator is Co-Chair of the European Association for Palliative Care Task Force *Wish to hasten death in patients with life-threatening illness: A clinical issue*, which comprises 26 researchers from 15 different institutions spread across 10 countries and three continents.

Team members have also shown leadership on the international stage, insofar as they were involved in research that led to an international consensus definition of the wish to hasten death and its related factors. As in the study proposed here, this research also made use of an international panel of experts and the Delphi technique. Finally, the team includes three senior researchers with expertise in conducting systematic and integrative reviews. The fact that the conceptual framework for developing the nursing diagnosis and its content will be derived from an umbrella review of systematic reviews brings rigour to the project and supports its validity.

### Timeline and expected results

Table 1 shows the research timeline and the expected results in each of the three phases.

**Table 1:** Phases of the research project, timeline, design and methodology, and expected results of each phase

Phase	Purpose of phase	Timeline	Research design and methodology	Expected results
**A**	Foundational knowledge	Nov 2023-Set 2024	Umbrella review of systematic reviews	The outcome of the review will provide the conceptual framework for developing the diagnosis and allow us to draw up a preliminary definition and set of defining characteristics and related factors
**B**	B.1. Concept analysis: Name of the diagnosis (label) based on the proposed definition	May–July 2024	Exploratory, descriptive, and diagnostic label study involving a panel of experts	Proposal of the most suitable label for the diagnosis, its definition, defining characteristics and related factors. This information will be collated in the form of a questionnaire comprising two sections: 1. The diagnostic label and its definition 2. Defining characteristics and related factors This questionnaire will then be used for the validation of diagnosis content (B.2)
	B.2. Validation of diagnosis content	Sept-November 2024	Delphi study to reach a consensus regarding the diagnostic label and its definition	Expert consensus regarding the diagnostic label and its definition
		Dec 2024-February 2025	Validation of diagnosis content using Fehring’s diagnostic content validation (DCV) model to determine the extent to which the defining characteristics and related factors are representative of the diagnosis	Measure of the extent to which each defining characteristic and related factor is representative of the diagnosis
				Indicator of overall level of confidence in the content of the diagnosis
**C**	To define nursing-specific interventions for the diagnosis	March-June 2025	C.1. Exploratory, descriptive and consensus study involving a panel of experts	Initial definition of nursing-specific interventions for responding to the wish to die
				Proposed interventions will be collated in the form of a questionnaire for use in a Delphi study
		July-Dec 2025	C.2. Exploratory, descriptive and consensus study using the Delphi technique	Expert consensus regarding nursing-specific interventions that can form part of the care plan for people with advanced disease who express the wish to die or hasten death

## Discussion

This study aims to define and validate a new nursing diagnosis for the wish to die among patients with advanced disease. However, the goal is not only to establish an expert consensus regarding the various components of this diagnosis (its label, definition, defining characteristics and related factors) but also to define and validate nursing-specific interventions for patients who express such a wish. This would be the first time that an attempt has been made to define an autonomous role for nurses in relation to exploring and responding clinically to the wish to die in end-of-life care. The identification and diagnosis of the wish to die in patients with advanced disease, and the application of defined nursing interventions will be useful for all countries, independently of its legislation regarding euthanasia. This phenomenon emerges as a reaction of suffering in patients with advanced disease; in this sense all patients could benefit from the detection of the phenomenon as well as from the implementation of the nursing-specific interventions for addressing the wish to die.

Our intention is that once the definitive version of the nursing diagnosis has been agreed (including not only its label and definition but also the defining characteristics and related factors), a formal submission would be made to NANDA, following the established protocol, requesting incorporation of the diagnosis into the NANDA-I taxonomy. One of the criteria for acceptance of a new diagnosis is that “the label and definition are distinct from other NANDA-I diagnoses and definitions” [13]. At present, neither the NANDA-I taxonomy nor the International Classification for Nursing Practice includes a diagnosis referring to the wish to die in the context of advanced disease, a wish that, as research has shown, has a multifactorial origin.

The method proposed here for defining and validating this new nursing diagnosis is consistent with the procedures recommended by NANDA and which have been used to date in developing its taxonomy. In this respect, it should be noted that the research team’s experience and knowledge regarding the wish to die among patients with advanced disease makes it ideally suited to carrying out this project. This also could be interpreted as a bias. In this way the project will include participants from different countries to avoid bias. In this way, the inclusion of nurses from different countries with or without euthanasia or MAiD legalized in nominal groups and Delphi technique (Phase B and C) will enrich the information for the definition of the new nursing diagnosis, the definition of its characteristics and related factors, and to define the nursing-specific interventions to be used in different cultural backgrounds.

Participants in Phase B could participate in Phase C; even though it could seem a limitation, the fact it is a strength because they are experts in with to die, and for this reason, the most appropriate participants to suggest nursing-specific interventions. In our opinion their participation is crucial in this Phase C of this project.

### Limitations

One limitation concerns the information that will be obtained from experts and how this will be used for validation purposes. Our application of the DCV model here relies on the retrospective opinions of nurses regarding the phenomenon of interest (the wish to die), and information will not be gathered in actual clinical situations [39]. Consequently, the proposed research will need to be complemented by clinical validation studies and studies examining construct validity. In this sense, the definition of nursing-specific interventions is also a limitation of this study. We will define nursing-specific intervention to address the wish to die, but we will not validate these interventions. Further clinical research will be needed to assess the impact and adequacy of these interventions.

Another limitation concerns the fact that all the experts will be recruited from within the field of palliative care. On the one hand, and given the focus and aims of the research, this might be considered a strength. Ideally, however, one would also include professionals whose work involves patients with chronic or neurological diseases, who may also experience a wish to die at some point in their process. A task for future research would therefore be to re-examine and validate the new nursing diagnosis with experts from other clinical fields and settings (e.g. long-stay facilities, primary care, neurology clinics).

### Impact for the nursing profession and conclusions

The proposed new nursing diagnosis would provide nurses with a standardised language and terminology regarding the wish to die in patients with advanced disease, and it would also facilitate communication about this phenomenon with professionals from other disciplines, as well as with patients and families. In addition, and together with the proposed nursing-specific interventions, it would encourage a more systematic approach to the exploration and treatment of the wish to die in the end-of-life setting, thereby paving the way for better and more individualised care plans.

### Supplementary Information

**Additional file 1.**

## Abbreviations

AFEDD: Assessment of the Frequency and Extent Desire to Die
DCV: Diagnostic content validation
MAiD: Medical assistance in dying
NANDA: North American Nurses Diagnosis Association
PRISMA: Preferred Reporting Items for Systematic Reviews and Meta-Analyses
WTHD: Wish to hasten death
