# Supplementary material for: Definition and validation of the nursing diagnosis label “wish to die”: a research protocol
Source: BMC Nurs. 2024 Jan 12;23:38. doi: 10.1186/s12912-024-01707-4 (PMC10785443; doi:10.1186/s12912-024-01707-4)
Supplement: Supplementary file 1 — Additional file 1. [file 12912_2024_1707_MOESM1_ESM.docx]

**Applying the NANDA-I taxonomy**

In the NANDA-I taxonomy, a diagnosis is constructed by combining the values of the three core axes, namely Axis 1 (the focus of the diagnosis), Axis 2 (subject of the diagnosis) and Axis 3 (judgment), and then adding, where relevant for clarity, the values of the other four axes (location, age, time and status of the diagnosis).

Following the recommendations of NANDA-I, this project will begin with the focus of the diagnosis (Axis 1) and then add the appropriate judgment term (Axis 3). As the subject of the diagnosis will always be an individual, Axis 2 is implicit and does not need to be included in the label.

Regarding Axis 1, the diagnosis *wish to die* will be proposed as a syndrome diagnosis, insofar as it is a clinical judgment related to a specific cluster of nursing diagnoses that occur together, and which are therefore best addressed together and through similar interventions (NANDA International, 2018)^[[1]](#footnote-1)^. In accordance with the theoretical framework described in the introduction to our proposal, and based on our clinical and research experience in relation to the wish to hasten death, we propose that the diagnosis could include diagnoses in the following NANDA-I domains:

- Domain 4: Activity/Rest
  - Class 2, Activity/exercise: Impaired physical mobility, impaired walking, impaired standing, etc.
  - Class 3, Energy balance: Fatigue.
  - Class 5, Self-care: Self-care deficit (feeding, bathing, toileting, etc.).
- Domain 6: Self-perception
  - Class 1, Self-concept: Risk for compromised human dignity, hopelessness.
  - Class 2, Self-esteem: Situational low self-esteem.
  - Class 3, Body image: Disturbed body image.
- Domain 7: Role relationship
  - Class 3, Role performance: Risk for ineffective role performance.
- Domain 9: Coping/Stress tolerance.
  - Class 2, Coping responses: Anxiety, death anxiety, fear.
- Domain 10: Life principles
  - Class 2, Beliefs: Spiritual distress, risk for impaired emancipated decision-making.
- Domain 12: Comfort
  - Class 1, Physical comfort: Pain.
  - Class 3, Social comfort: Social isolation.

This is the initial rationale for why the proposed diagnosis would correspond to a syndrome diagnosis. However, this aspect would be analysed in depth as part of the project we are proposing.

1. NANDA International. (2018). *Nursing diagnoses: Definitions and classification 2018-2020*. 11th edition, edited by T. H. Herdman & S. Kamitsuru. Thieme: New York. [↑](#footnote-ref-1)
